# Postoperative onset lateral hinge fracture is a risk factor for delayed union of the tibial tuberosity in medial opening wedge distal tibial tuberosity osteotomy

**DOI:** 10.1016/j.asmart.2024.01.005

**Published:** 2024-07-02

**Authors:** Hiroyasu Ogawa, Yutaka Nakamura, Masaya Sengoku, Tetsuya Shimokawa, Kazuichiro Ohnishi, Haruhiko Akiyama

**Affiliations:** a Department of Orthopaedic Surgery, Gifu University Graduate School of Medicine, Yanagido 1-1, Gifu, 501-1194, Japan; b Department of Orthopaedic Surgery, Ogaki Tokushukai Hospital, Hayashi-machi 6-85-1, Ogaki, Gifu, 503-0015, Japan

**Keywords:** Distal tibial tuberosity osteotomy, OWDTO, High tibial osteotomy, Bone union, Risk factors, Lateral hinge fracture

## Abstract

**Background:**

This study aimed to evaluate bone union of the tibial tuberosity in patients undergoing medial opening wedge distal tibial tuberosity osteotomy (OWDTO). It was hypothesized that bone union of the tibial tuberosity could be associated with lateral hinge fractures (LHFs), but not thickness of the tibial tuberosity osteotomy.

**Methods:**

Data of 61 consecutive patients who underwent OWDTO were retrospectively reviewed. Radiographic parameters of the lower limb and LHFs were evaluated. Thickness of the tibial tuberosity osteotomy and bone union of the tibial tuberosity were assessed at 1, 2, 3, 4, and 5 cm distal to the most proximal part of the tibial tuberosity on computed tomography. Bone union was assessed. Factors related to bone union of the tibial tuberosity were analyzed.

**Results:**

There were 13 postoperative onset LHFs: all healed with conservative treatments within 6 months after surgery. The total score of bone union of the tibial tuberosity was 8.4 ± 2.1 points, which correlated with age, postoperative medial proximal tibial angle (MPTA), correction angle, and postoperative onset LHF (r = 0.307, 0.388, 0.275, and −0.624, respectively; *p* = 0.016, 0.002, 0.033, and <0.001, respectively). Regression coefficient for postoperative onset LHF, postoperative MPTA, and body mass index were −0.619 (*p* < 0.001), 0.285 (*p* = 0.003), and −0.227 (*p* = 0.021), respectively.

**Conclusion:**

Postoperative onset LHFs, but not thickness of the tibial tuberosity osteotomy, were a risk factor for delayed union of the tibial tuberosity following OWDTO. Furthermore, to prevent delayed union of the tibial tuberosity, postoperative onset LHFs should be prevented.

**Level of evidence:**

LEVEL III, Case-control study.

## 1 Introduction

Medial opening wedge distal tibial tuberosity osteotomy (OWDTO), which protects against progression of cartilage degeneration in the patellofemoral joint, can be an alternative to medial opening wedge high tibial osteotomy (MOWHTO).^1^, ^2^, ^3^, ^4^, ^5^ However, OWDTO has potential risk of tibial tuberosity osteotomy-related complications, including tibial tuberosity fractures and delayed- or non-union of the tibial tuberosity.^6^^,^^7^

As delayed- or non-union of the tibial tuberosity may cause knee pain, decreased knee function, and further tibial tuberosity fractures,^7^ their prevention is crucial for patients undergoing OWDTO. Generally, the bone union rate is defined by the area of osteotomy and the type of bone compartment (cancellous or cortical compartment).^8^, ^9^, ^10^ As the distal tibial tuberosity has a relatively high proportion of cortical bone, bone union of the tibial tuberosity in patients undergoing OWDTO is concerning.^6^ However, there is a scarcity of literature on bone union of the tibial tuberosity in patients undergoing OWDTO and its related factors.

This study aimed to assess bone union of the tibial tuberosity in patients undergoing OWDTO and its related factors, according to bone compartments. It was hypothesized that bone union of the tibial tuberosity can be associated with lateral hinge fractures (LHFs), but not thickness of the tibial tuberosity osteotomy because the tibial tuberosity, except the most distal part, is cancellous in nature.

## 2 Materials and methods

### 2.1 Patient selection

Sixty-one consecutive patients who underwent OWDTO between April 2020 and March 2021 at our institution were retrospectively evaluated. During this period, conventional MOWHTO was not performed.^11^ All surgeries were performed by a single surgeon. The surgical indications for OWDTO were medial compartment knee osteoarthritis (OA) and impaired performance of daily living activities due to knee pain that persisted despite conservative treatment for at least 3 months.^4^^,^^12^ The exclusion criteria were: anterior cruciate ligament (ACL) deficiency, flexion contracture >10°, and hip-knee-ankle (HKA) angle >15°.^13^, ^14^ Average age of the patients was 58.0 ± 7.7 years and 36 participants (59.0%) were females. The average body mass index was 27.2 ± 3.9 kg/m^2^, and the average Kellgren–Lawrence grade was 2.8 ± 0.6. The patient follow-up rate was 100% for clinical assessment and imaging evaluation.

### 2.2 Surgical procedures and postoperative rehabilitation

OWDTO was performed as previously described.^4^^,^^6^ First, distal tibial tuberosity osteotomy was performed parallel to the tibial bone axis such that the distal tibial tuberosity osteotomy was 7–8 mm in thickness, at the thickest part, and approximately 5 cm in length. Second, transverse osteotomy was performed perpendicular to the tibial bone axis. The tibia was fixed with a Tris Medial HTO Plate System and Osferion60 β-tricalcium phosphate wedge spacers (Olympus Terumo Biomaterials, Tokyo, Japan). Finally, the tibial tuberosity was fixed with cannulated partially threaded screws to the posterior tibial cortex. The numbers of screws were one and two in female and male patients, respectively.

In the postoperative rehabilitation program, the range of motion exercises were started a few days post-surgery, and the patients were allowed half and full weight-bearing in the second and third weeks, respectively.^15^^,^^16^

### 2.3 Assessment of lower limb alignment, valgus correction, and LHFs

The HKA angle, medial proximal tibial angle (MPTA), mechanical lateral distal femoral angle, and posterior tibial slope (PTS) were measured using computed radiography (CR).^16^^,^^17^ The valgus correction angle and gap opening distance were measured based on postoperative computed tomography (CT) scans. LHFs were assessed on CT scans one week post-surgery, as well as on CR at 3 weeks, and 2, 3, and 6 months postoperatively.^18^ Tibial tuberosity fractures were also assessed using CT scans one week after surgery. LHFs that were not recognized on CT scans one week post-surgery, but diagnosed thereafter based on CR images, were defined as postoperative onset LHFs.

### 2.4 Assessment of the tibial tuberosity

**Fig. 1:**
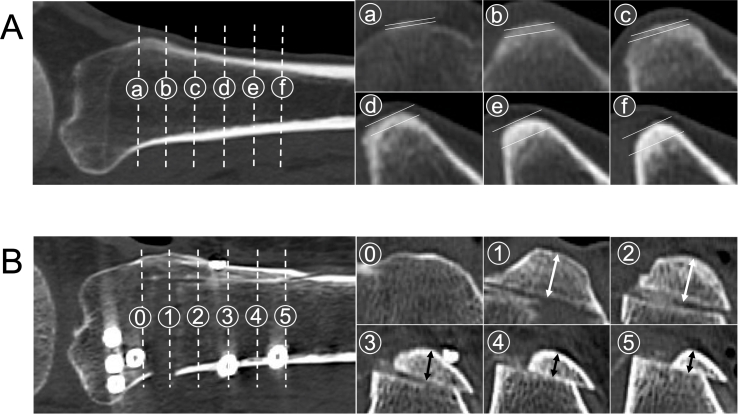
Assessment of the tibial tuberosity. A) Preoperative CT scans of the proximal tibia. ⓐ, ⓑ, ⓒ, ⓓ, ⓔ, and ⓕ indicate 0, 1, 2, 3, 4, and 5 cm from the most proximal part of the tibial tuberosity. The distance between two white lines indicates the thickness of the cortical compartment of the tibial tuberosity. B) CT scans of the proximal tibia 3 days after surgery. ⓪, ①, ②, ③, ④, and ⑤ indicate 0, 1, 2, 3, 4, and 5 cm from the most proximal part of the tibial tuberosity. White or black arrows indicate the thickness of the tibial tuberosity osteotomy.

The cortical compartment thickness of the tibial tuberosity was measured 1, 2, 3, 4, and 5 cm distal to the most proximal part of the tibial tuberosity on the axial views of preoperatively obtained CT scans (Fig. 1A). Thickness and bone union of the tibial tuberosity were similarly assessed on CT scans 1 week and 6 months after surgery, respectively (Fig. 1B). Bone union was scored as 0, 1, and 2 points for non-union, partial union, and complete union, respectively, at the five locations, and thus the total point ranges were between 0 and 10 points.

### 2.5 Clinical evaluation

Clinical symptoms were evaluated by the pain visual analog scale (VAS) at rest and during motion preoperatively, as well as at 3 weeks and 2, 3, and 6 months post-surgery. The Knee Society Score (KSS) was also used, comprising the total knee and functional scores, preoperatively and at 6 months and 1 year post-surgery.

### 2.6 Reliability for CT scan measurement of the tibial tuberosity

The tibial tuberosity cortical compartment thickness and bone union of the tibial tuberosity were measured on CT scans by two orthopedic surgeons twice, and the mean values were used for analysis (Fig. 1). The intra- and inter-measurer reliabilities of the measurements were expressed using the intraclass correlation coefficients. The intra- and inter-measurer reliabilities of the tibial tuberosity cortical compartment thickness were 0.89 and 0.85, respectively, and those of the tibial tuberosity bone union were 0.90 and 0.89, respectively.

### 2.7 Statistical analysis

GraphPad Prism version 9.0 (GraphPad Software, La Jolla, CA, USA) and SPSS version 13.0 (IBM Corp., Armonk, NY, USA) were used for statistical analyses. Preoperative and postoperative radiographic parameters (HKA angle, MPTA, and PTS) were compared using a paired *t*-test. In terms of KSS and pain VAS, analysis of multiple points was performed using the one-way ANOVA with Tukey's post-hoc test. Spearman's correlation analysis was performed to assess the relationship between the total point of the bone union score and other variables (age, female sex, body mass index, postoperative HKA angle, postoperative MPTA, postoperative PTS, correction angle, gap opening distance, tibial cortical component thickness, tibial tuberosity osteotomy thickness, postoperative onset LHF, pain VAS, and KSS). Multiple regression analysis using stepwise selection was performed to determine the risk factors for delayed union of the tibial tuberosity. The dependent variable was the total point of the bone union score. Additionally, the independent variables were age, sex, body mass index, postoperative HKA angle, postoperative MPTA, postoperative PTS, correction angle, gap opening distance, average tibial tuberosity thickness, average tibial tuberosity osteotomy thickness, and postoperative onset LHF. P-values less than 0.05 and regression coefficients (B) with a 95% confidence interval that did not include 1 were considered significant.

## 3 Results

### 3.1 Lower limb alignment and complications

Data including the HKA angle, MPTA, and PTS are summarized in Table 1. There were 13 postoperative onset LHFs of Takeuchi type I, all of which healed with conservative treatments within 6 months post-surgery, and no incidence of delayed- or non-union was observed. No instances of complications or reoperations, whether or not related to the tibial tuberosity, were observed.

**Table 1:** Radiographic parameters (n = 61).

HKA angle^b^, degree, mean ± SD	Preoperative	5.6 ± 2.9
	Postoperative	−3.7 ± 2.1^a^
MPTA, degree, mean ± SD	Preoperative	84.5 ± 2.8
	Postoperative	93.6 ± 1.6^a^
PTS, degree, mean ± SD	Preoperative	9.4 ± 2.7
	Postoperative	9.2 ± 2.9
Correction angle, degree, mean ± SD		9.3 ± 2.0
Gap opening distance, mm, mean ± SD		10.6 ± 2.6
Postoperative lateral hinge fracture, n (%)		13 (21.3)

HKA angle, hip–knee–ankle angle; MPTA, medial proximal tibial angle; PTS, posterior tibial slope; SD, standard deviation.

a p < 0.001 compared with the preoperative value, paired *t*-test.

b A positive value indicates varus deformity.

### 3.2 Thickness of the tibial tuberosity cortical compartment and osteotomy

Data of the thickness of the tibial tuberosity cortical compartment and tibial tuberosity osteotomy is presented in Table 2. The tibial tuberosity cortical compartment became thicker towards the distal side, and the thickest and thinnest parts were 7.3 ± 1.9 mm and 3.4 ± 1.4 mm at 2 cm and 5 cm distal to the most proximal part of the tibial tuberosity, respectively. The thickness of the tibial tuberosity osteotomy was greater than that of the cortical compartment of the tibial tuberosity at 1–4 cm distal to the most proximal point of the tibial tuberosity.

**Table 2:** Thickness of the cortical compartment of the tibial tuberosity and tibial tuberosity osteotomy (n = 61).

Thickness, mm, mean ± SD	Distal to the most proximal part of the tibial tuberosity					
	1 cm	2 cm	3 cm	4 cm	5 cm	Average
Tibial tuberosity cortical compartment	3.1 ± 1.1	3.9 ± 1.4	4.0 ± 1.3	4.0 ± 1.5	4.2 ± 1.6	3.9 ± 1.0
Tibial tuberosity osteotomy	6.1 ± 1.9	7.3 ± 1.9	6.3 ± 1.8	4.6 ± 1.5	3.4 ± 1.4	5.6 ± 1.4

SD, standard deviation.

### 3.3 Bone union after tibial tuberosity osteotomy

The scores of bone union following tibial tuberosity osteotomy are summarized in Table 3. The total score was 8.4 ± 2.1 points. Although the bone union score tended to decrease towards the distal side, this score was consistently high over the entire range.

**Table 3:** CT scan evaluation of bone union of the tibial tuberosity osteotomy 6 months after OWDTO (n = 61).

Distance from the most proximal part of the tibial tuberosity	1 cm	2 cm	3 cm	4 cm	5 cm	Total point
Bone union score^a^, point, mean ± SD	1.9 ± 0.3	1.8 ± 0.5	1.7 ± 0.5	1.6 ± 0.7	1.4 ± 0.8	8.4 ± 2.1

a Bone union score: 2 points for complete union, 1 point for incomplete union, and 0 point for non-union; SD, standard deviation.

### 3.4 KSS and pain VAS

**Fig. 2:**
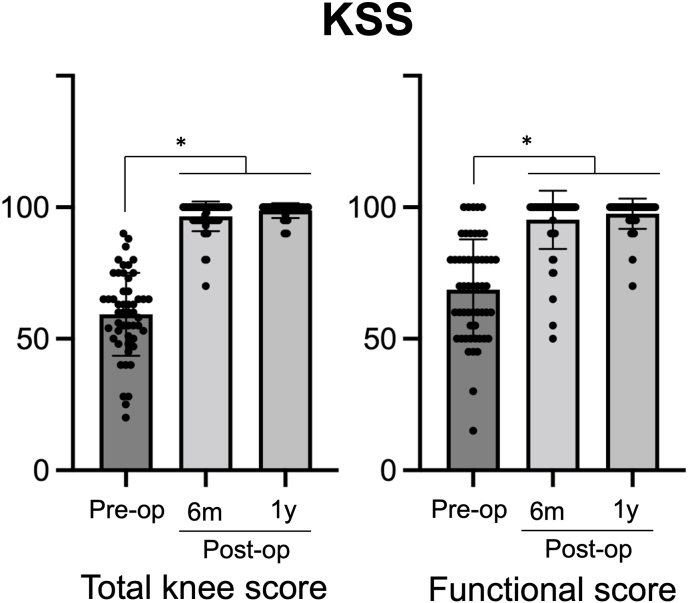
KSS (Total knee score and functional score) at 6 months and one year after OWDTO.

**Fig. 3:**
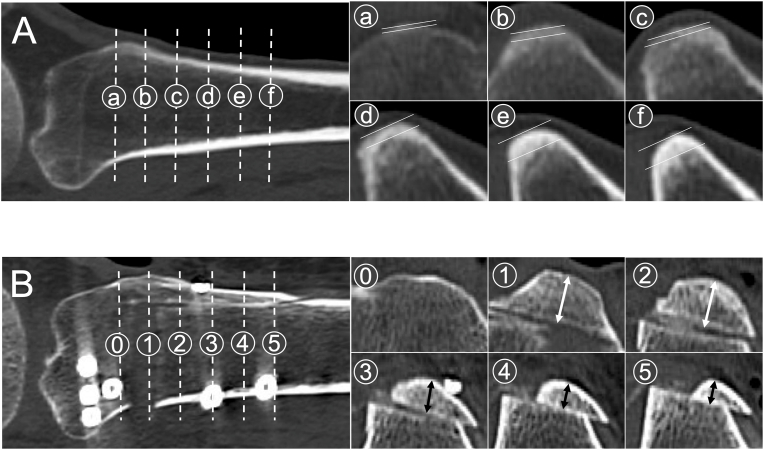
Pain VAS at 6 months and one year after OWDTO.

Both, total knee and functional scores significantly improved at 6 months post-surgery (*p* < 0.0001 for each comparison, Fig. 2). Preoperatively, the total knee and functional scores were 59.3 ± 15.8 and 68.7 ± 19.1, respectively. Post-surgery, the total knee and functional scores were 96.5 ± 5.6 and 95.3 ± 11.1 at 6 months and 98.8 ± 2.8 and 97.6 ± 5.8 at 1 year, respectively. All knee score components, except flexion contracture (pain, extension lag, total range of flexion, and alignment), improved significantly post-surgery (p = 0.035 for total range of flexion, p < 0.001 for the others). All functional components, except for walking aids used, improved significantly post-surgery (p < 0.001 for all comparison). Pain VAS score during motion significantly decreased at 2 months following OWDTO (*p* < 0.0001, Fig. 3).

### 3.5 Factors correlated with bone union after the tibial tuberosity osteotomy

Spearman's correlation analysis was performed to determine the related factors for bone union of the tibial tuberosity (Table 4). The total point of the bone union score significantly correlated with age, postoperative MPTA, correction angle, and postoperative onset LHF (r = 0.307, 0.388, 0.275, and −0.624, respectively; *p* = 0.016, 0.002, 0.033, and <0.001, respectively).

**Table 4:** Factors related to bone union of the tibial tuberosity osteotomy (n = 61).

Variable	Correlation coefficient	p-value
Age	0.307	0.016
Female sex	0.257	n.s.
Body mass index	−0.029	n.s.
Postoperative HKA angle	0.097	n.s.
Postoperative MPTA	0.388	0.002
Postoperative PTS	0.078	n.s.
Correction angle	0.275	0.033
Gap opening distance	0.153	n.s.
Tibial cortical component thickness	0.121	n.s.
Tibial tuberosity osteotomy thickness	0.192	n.s.
Postoperative lateral hinge fracture	−0.624	<0.001

HKA angle, hip–knee–ankle angle; MPTA, medial proximal tibial angle; PTS, posterior tibial slope; n.s., not significant.

### 3.6 Regression coefficients for bone union of the tibial tuberosity

Regression coefficients were calculated to assess the influence of the tibial tuberosity on bone union using a multiple regression analysis (Table 5). The regression coefficients for postoperative onset LHF, postoperative MPTA, and body mass index were −0.619 (*p* < 0.001), 0.285 (*p* = 0.003), and −0.227 (*p* = 0.021), respectively.

**Table 5:** Regression coefficients for bone union of the tibial tuberosity (n = 61).

Variable	β^a^	B^b^	Std. Err. of B	95 % CI of B	t-value	p-value
Postoperative lateral hinge fracture	−0.619	−3.155	0.486	−4.129–2.181	−6.495	<0.001
Postoperative MPTA	0.285	0.054	0.018	0.019–0.089	3.067	0.003
Body mass index	−0.227	−0.128	0.054	−0.236–0.020	−2.377	0.021

MPTA, medial proximal tibial angle; CI, confidence interval.

a Standardized regression coefficients.

b Unstandardized coefficients.

### 3.7 Correlation between bone union of the tibial tuberosity and clinical evaluation

The total score of bone union of tibial tuberosity osteotomy was not statistically correlated with the total knee and pain VAS scores at 3 weeks and 2, 3, and 6 months post-surgery. However, it correlated with the functional score at 6 months (r = 0.279, *p* = 0.028) and 1 year (r = 0.422, *p* = 0.004) post-surgery (Table 6).

**Table 6:** Correlation between bone union of the tibial tuberosity osteotomy and the KSS (n = 61).

KSS		Correlation coefficient	p-value
Total knee score	6 months	0.216	n.s.
	1 year	0.091	n.s.
Functional score	6 months	0.279	0.038
	1 year	0.422	0.004

KSS, Knee Society Score; n.s., not significant.

### 3.8 Bone union of the tibial tuberosity and tibial tuberosity osteotomy thickness

The tibial tuberosity osteotomy thickness was not found to be associated with bone union of the tibial tuberosity in patients undergoing OWDTO.

## 4 Discussion

The most important findings of the present study indicated that postoperative onset LHFs, postoperative MPTA, and body mass index, but not the thickness of the tibial tuberosity osteotomy, were risk factors for delayed union of the tibial tuberosity following OWDTO. In addition, bone union of the tibial tuberosity was associated with clinical outcomes 1 year post-surgery. Bone union of the tibial tuberosity may be a concern following OWDTO because the tibial tuberosity seems to be unfavorable to bone union due to a high portion of cortical compartment and pull-out force to the tibial tuberosity by transmission of the quadriceps muscle strength.^4^^,^^5^^,^^19^^,^^20^ The results in the current study revealed that for good bone union of the tibial tuberosity following OWDTO, attention should be paid to postoperative onset LHFs, postoperative MPTA, and body mass index, and not to the thickness of the tibial tuberosity osteotomy. However, there is no significant difference in LHF incidence occurrence between OWDTO and conventional MOWHTO.^21^ This study makes a novel contribution to the literature as it shows the relationship between bone union of the tibial tuberosity after OWDTO and postoperative onset LHFs.

Although bone union may be facilitated by a thicker tibial tuberosity osteotomy, a recent biomechanical study by Ogawa et al. showed that a thinner distal tibial tuberosity was more resistant to axial load at the tibial shaft and lateral hinge. They also concluded that a thin distal tibial tuberosity osteotomy may be recommended for the prevention of tibial and lateral hinge fractures after OWDTO.^19^ Furthermore, some studies have shown good clinical outcomes and bone union of the tibial tuberosity following OWDTO with 7–8 mm thick tibial tuberosity osteotomies.^4^^,^^6^^,^^15^ Thus, the thickness and osteotomy method should be determined from the view of bone union and mechanical strength around osteotomy. In the current study, the thickness of the tibial tuberosity cortical compartment and tibial tuberosity osteotomy were identified on CT scans. When the thickness of the tibial tuberosity osteotomy was 7.3 ± 1.9 mm at the thickest part, the osteotomy went through the cancellous compartment, 4 cm distal to the most proximal part of the tibial tuberosity, and was cut into the tibial tuberosity cortical compartment at 5 cm. Although the rate of bone union after the tibial tuberosity osteotomy decreased on moving distally, the thickness of the tibial tuberosity did not correlate with bone union.

Most importantly, a postoperative onset LHF was one of the risk factors for delayed union of the tibial tuberosity, suggesting that LHFs destabilize the tibial tuberosity osteotomy site following OWDTO, as they delay healing of the osteotomy gap following conventional MOWHTO.^18^^,^^22^ The pull-out force rather than compressive force may affect bone union of the tibial tuberosity by transmission of the quadriceps femoris strength after OWDTO. The reason for the postoperative MPTA to be a risk factor for delayed union could be that the tibial tuberosity flange can slide more medially in the case of a large postoperative MPTA, resulting in reduced contact area between the tibial tuberosity and distal fragment of the tibia.^8^ Another possible explanation is that large correction followed by large MPTA may induce more stress on tibial tuberosity osteotomy site as well as lateral hinge points. Therefore, MPTA may be associated with bone union of the tibial tuberosity more than only correction angle. The knee functional score at 1 year post- OWDTO was significantly associated with bone union of the tibial tuberosity in contrast to the pain VAS score. As bone union strongly correlates with the incidence of postoperative onset LHFs, the cases of delayed union of the tibial tuberosity were likely influenced by postoperative onset LHFs, which might have been the main cause of the decreased knee functional score. Prevention of postoperative onset LHFs is important for bone union following tibial tuberosity osteotomy. The results in this study suggested that postoperative weight bearing may be delayed for prevention of postoperative onset LHFs, particularly in patients with high postoperative MPTA and body mass index.^23^

In terms of clinical outcomes, KSS at 6 months and 1 year post-surgery was significantly higher than that preoperatively and comparable to that in previous reports.^4^^,^^6^ Of note, delayed union of the tibial tuberosity correlated with KSS functional score till 1 year post-surgery (Table 6). As the functional scores at 6 months and 1 year post-surgery significantly correlates with bone union of the tibial tuberosity, delayed union might affect elements of walking and stair climbing. Therefore, it is important to take care of bone union of the tibial tuberosity. This finding in the current study differs from that of a previous study by Han et al., wherein there were no significant differences in postoperative KSS following MOWHTO at 6 months and 1 year post-surgery, based on the presence of LHFs.^23^ Any non-significant difference may have been due to variation in the type of LHF, intraoperative or postoperative; postoperative onset LHFs are usually diagnosed at 2–3 months after surgery and subsequently may delay treatment.

This study has some limitations. The possibility of postoperative onset LHF occurrence during surgery cannot be ruled out. The mechanism of LHFs leading to delayed union of the tibial tuberosity remains unknown. In addition, the impact of delayed union of the tibial tuberosity and postoperative onset LHFs on knee score should have been assessed.

## 5 Conclusion

An advantage of OWDTO over MOWHTO is prevention of patellofemoral joint degeneration; therefore, it is an alternative procedure for medial compartment osteoarthritis of the knee. However, surgeons should pay close attention to postoperative onset LHF occurrence, even if it is not recognized radiographically immediately after surgery. The risk factors for delayed union of the tibial tuberosity were postoperative onset LHFs, postoperative MPTS, and body mass index. These results are clinically relevant for surgeons performing OWDTO. To avoid delayed union of the tibial tuberosity, postoperative onset LHFs should be prevented.

## Funding

There was no external funding for this study.

## Ethical approval

This study was approved by the Institutional Review Board of the author's institution (approval number: TGE01668-066).

## Disclosure of interest

The authors have no potential conflicts of interest to disclose in relation to this study, including financial interests, activities, relationships, and affiliations.

## Authors' contribution

Hiroyasu Ogawa: Study design, data collection and analysis, and manuscript drafting. Masaya Sengoku, Yutaka Nakamura, Tetsuya Shimokawa, and Kazuichiro Ohnishi: Collection and analysis of data, drafting the manuscript. Haruhiko Akiyama: drafting the manuscript.

## Declaration of competing interest

The authors have no conflicts of interest relevant to this article.

## References

[1] Kim K.I., Kim D.K., Song S.J., Lee S.H., Bae D.K. (2017). Medial open-wedge high tibial osteotomy may Adversely affect the patellofemoral joint. Arthroscopy.

[2] Goshima K., Sawaguchi T., Shigemoto K., Iwai S., Nakanishi A., Ueoka K. (2017). Patellofemoral osteoarthritis progression and alignment changes after open-wedge high tibial osteotomy Do not affect clinical outcomes at Mid-term follow-up. Arthroscopy.

[3] Krause M., Drenck T.C., Korthaus A., Preiss A., Frosch K.H., Akoto R. (2018). Patella height is not altered by descending medial open-wedge high tibial osteotomy (HTO) compared to ascending HTO. Knee Surg Sports Traumatol Arthrosc.

[4] Ogawa H., Matsumoto K., Yoshioka H., Sengoku M., Akiyama H. (2020). Distal tibial tubercle osteotomy is superior to the proximal one for progression of patellofemoral osteoarthritis in medial opening wedge high tibial osteotomy. Knee Surg Sports Traumatol Arthrosc.

[5] Gaasbeek R.D., Sonneveld H., van Heerwaarden R.J., Jacobs W.C., Wymenga A.B. (2004). Distal tuberosity osteotomy in open wedge high tibial osteotomy can prevent patella infera: a new technique. Knee.

[6] Ogawa H., Matsumoto K., Yoshioka H., Sengoku M., Akiyama H. (2020). Fracture of the tibial tubercle does not affect clinical outcomes in medial opening wedge high tibial osteotomy with distal tibial tubercle osteotomy. Arch Orthop Trauma Surg.

[7] Kuwashima U., Itoh M., Itou J., Okazaki K. (2022). Tibial shaft fracture after medial open-wedge distal tibial tuberosity osteotomy: a case report. Clin Case Rep.

[8] Nejima S., Kumagai K., Fujimaki H. (2021). Increased contact area of flange and decreased wedge volume of osteotomy site by open wedge distal tibial tuberosity arc osteotomy compared to the conventional technique. Knee Surg Sports Traumatol Arthrosc.

[9] Johnson K.D., August A., Sciadini M.F., Smith C. (1996). Evaluation of ground cortical autograft as a bone graft material in a new canine bilateral segmental long bone defect model. J Orthop Trauma.

[10] Kim J.K., Yoon J.O., Baek H. (2018). Corticocancellous bone graft vs cancellous bone graft for the management of unstable scaphoid nonunion. Orthop Traumatol Surg Res.

[11] Staubli A.E., De Simoni C., Babst R., Lobenhoffer P. (2003). TomoFix: a new LCP-concept for open wedge osteotomy of the medial proximal tibia--early results in 92 cases. Injury.

[12] Horikawa T., Kubota K., Hara S., Akasaki Y. (2020). Distal tuberosity osteotomy in open-wedge high tibial osteotomy does not exacerbate patellofemoral osteoarthritis on arthroscopic evaluation. Knee Surg Sports Traumatol Arthrosc.

[13] Kim K.I., Kim G.B., Kim H.J., Song S.J. (2018). Does the pre-operative status of the anterior cruciate ligament affect the outcomes following medial open-wedge high tibial osteotomy?. Knee.

[14] Ogawa H., Matsumoto K., Yoshioka H., Sengoku M., Akiyama H. (2019). Distal tibial tubercle osteotomy is superior to the proximal one for progression of patellofemoral osteoarthritis in medial opening wedge high tibial osteotomy. Knee Surg Sports Traumatol Arthrosc.

[15] Ogawa H., Matsumoto K., Sengoku M. (2021). Clinical course and outcomes of simultaneous-versus staged-bilateral medial opening wedge high tibial osteotomy. Asia Pac J Sports Med Arthrosc Rehabil Technol.

[16] Ogawa H., Matsumoto K., Ogawa T., Takeuchi K., Akiyama H. (2016). Preoperative varus laxity correlates with overcorrection in medial opening wedge high tibial osteotomy. Arch Orthop Trauma Surg.

[17] Ogawa H., Matsumoto K., Ogawa T., Takeuchi K., Akiyama H. (2016). Effect of wedge insertion angle on posterior tibial slope in medial opening wedge high tibial osteotomy. Orthop J Sports Med.

[18] Ogawa H., Matsumoto K., Akiyama H. (2017). The prevention of a lateral hinge fracture as a complication of a medial opening wedge high tibial osteotomy: a case control study. Bone Joint Lett J.

[19] Ogawa H., Nakamura Y., Sengoku M. (2022). Thinner tuberosity osteotomy is more resistant to axial load in medial open-wedge distal tuberosity proximal tibial osteotomy: a biomechanical study. Knee.

[20] Akiyama T., Osano K., Mizu-Uchi H. (2019). Distal tibial tuberosity arc osteotomy in open-wedge proximal tibial osteotomy to prevent patella infra. Arthrosc Tech.

[21] Ogawa H., Nakamura Y., Matsumoto K., Akiyama H. (2023). Incidence and risk factors for lateral hinge fractures in medial opening wedge high tibial osteotomy and medial opening wedge distal tibial tuberosity osteotomy. Knee.

[22] Kumagai K., Yamada S., Nejima S., Muramatsu S., Akamatsu Y., Inaba Y. (2020). Lateral hinge fracture delays healing of the osteotomy gap in opening wedge high tibial osteotomy with a beta-tricalcium phosphate block. Knee.

[23] Han S.B., Choi J.H., Mahajan A., Shin Y.S. (2019). Incidence and predictors of lateral hinge fractures following medial opening-wedge high tibial osteotomy using locking Plate System: better performance of computed tomography scans. J Arthroplasty.

